# MOF-Derived MnO/C Nanocomposites for High-Performance Supercapacitors

**DOI:** 10.3390/nano12234257

**Published:** 2022-11-30

**Authors:** Yuqing Qiao, Na Li, Mingwei Dong, Peng Jia, Chongchong Ma, Tong Zhang, Tifeng Jiao

**Affiliations:** 1 Hebei Key Laboratory of Applied Chemistry, School of Environmental and Chemical Engineering, Yanshan University, Qinhuangdao 066004, China; 2 State Key Laboratory of Metastable Materials Science and Technology, Yanshan University, Qinhuangdao 066004, China

**Keywords:** metal–organic frameworks, manganese monoxide, nanocomposite, supercapacitor

## Abstract

As ordered porous materials, metal–organic frameworks (MOFs) have attracted tremendous attention in the field of energy conversion and storage due to their high specific surface area, permanent porosity, and tunable pore sizes. Here, MOF-derived MnO/C nanocomposites with regular octahedral shape were synthesized using a Mn-based analogue of the MIL-100 framework (Mn-MIL-100, MIL: Matérial Institut Lavoisier) as the precursor. Using aberration-corrected environmental transmission electron microscopy (ETEM), MnO nanocages with a diameter of approximately 20 nm were recognized in the MnO/C nanocomposites fabricated, dispersed in a microporous carbon matrix homogeneously. The nanocages are composed of MnO nanoparticles with a diameter of approximately 2 nm and with a single crystal structure. The specific surface area of the as-prepared MnO/C octahedra decreases to 256 m^2^ g^−1^ from 507 m^2^ g^−1^ of the Mn-MIL-100 precursor, whereas the total pore volume increases to 0.245 cm^3^ g^−1^, which is approximately 29% higher than that of the precursor (0.190 cm^3^ g^−1^). Additionally, when utilized as an electrode for supercapacitors, the MOF-derived MnO/C nanocomposite demonstrates a towering specific capacitance of 421 F g^−1^ at 0.5 A g^−1^ and good cycle stability (94%) after 5000 cycles. Our work reveals that the MnO nanoparticles in MOF-derived MnO/C nanocomposites exhibit nanocage structure characteristics, which might be inherited from the Mn-MIL-100 precursor with analogous supertetrahedron units.

## 1. Introduction

In the field of renewable energy sources, synthesizing electrode materials with both high energy density and high power density is critical to practical applications [1]. Supercapacitors, known as electrochemical capacitors, can be classified as electrical double-layer capacitors (EDLC) and pseudo-capacitors according to the charge-storage mechanisms. The electrode materials of EDLC can adsorb and store electrolyte ions on their surface without redox reactions, resulting in good cycle stability and high power [2,3]. Pseudo-capacitance is used to designate electrode materials, showing an electrochemical signature with capacitive electrode: a linear relationship between the charge stored and Faradaic reaction mechanisms at the electrode materials. The pseudo-capacitors have a higher energy density and capacitance than the carbon electrode materials used in EDLC. In the last few years, some investigations have been focused on composite electrode materials combining the two charge-storage mechanisms. This is because the composite electrode materials with both a porous structure and a high surface area should be beneficial to maintaining high specific capacitance coupled with good cycle stability [4,5]. MOFs have been extensively used as precursors to design porous carbons and carbon-based composites [6,7,8,9,10,11,12,13]. As one of the most competitive candidate electrode materials of pseudo-capacitors, manganese oxides, including manganese oxides derived from MOFs, have been prepared with good electrochemical performance [14,15,16,17,18,19]. However, like most metal oxides, the low electronic conductivity, the limited effective area, and the rapid capacity fading resulting from the volume changes and the agglomeration of manganese oxide active materials area major challenge to the improved electrochemical performance of manganese oxide electrode materials.

As a class of sophisticated nanostructured materials, MOFs have been extensively used for designing and fabricating porous composites with tunable pore sizes and high performance [20,21,22,23,24,25]. Li et al. calcined hexagonal cuboid Co-MOF-74 crystals at an optimized temperature of 350 °C to prepare porous Co_3_O_4_ nanotubes, which revealed a high specific capacitance of 647 F g^−1^ with a current density of 1 A g^−1^. The Co_3_O_4_ nanotubes were used as electrode materials for supercapacitors [26]. Zheng et al. prepared MOF-derived MnO nanoparticles distributed in a porous carbon matrix homogeneously. As an electrode material for lithium-ion batteries, the MnO/C exhibited an excellent capacity of 1221 mAh g^−1^ at a current density of 1 A g^−1^ after 100 cycles [11]. Zhang et al. prepared MOF-derived porous Mn_2_O_3_ octahedra with heat treatment of the Mn-MIL-100 precursors at 400 °C, and the MOF-derived Mn_2_O_3_ octahedra electrode materials revealed a high capacity of 755 mAh g^−1^ after 100 cycles [27].

In addition, Zhang et al. obtained irregular cubic porous Mn_2_O_3_ by heat treatment of the Mn-MIL-100 precursors at 700 °C, which revealed intensive catalytic activity for CO oxidation [15]. They also demonstrated that the calcination temperature for the MOF precursors had an obvious effect on the catalytic activity of the Mn_2_O_3_ prepared. Reinsh et al. prepared an Mn-based analogue of the MIL-100 framework, and the structure of the obtained MOF was revealed by time-dependent energy-dispersive X-ray powder diffraction (EDXPD) experiments carried out under solvothermal conditions [14]. The results showed that the framework structure of the obtained MOF was stable in both methanol and in ethanol, whereas the crystallinity decreased when the solvent was exchanged with water. In addition, the MOF revealed an amorphous structure when those solvents were removed by heating under vacuum, whereas a crystal structure was obtained when those solvents were removed at room temperature under vacuum. Furthermore, Reinsh et al. reported that the residual acid can be removed easily by washing the synthesized MOF in ethanol solution, which is necessary for the MOF to remove the unreacted organic linkers, as the residual acid might be attached or encapsulated within the MOF.

Generally, MOFs are fabricated by metal ion units and organic linkers [14,28,29]. G Férey et al. reported a new crystalline MOF, MIL-100, with a very high surface area up to 3100 m^2^ g^−1^, which is configured by a supertetrahedron with inorganic bricks as the corners and tricarboxylate anions as the faces [29]. Two types of cavities are fabricated from the cages of the supertetrahedron—pentagonal windows and hexagonal apertures—and two types of pores are obtained with a diameter of approximately 2.5 nm and 2.9 nm, respectively. Although it is a challenge to observe the nanocages or the topological structure of MOFs in TEM due to the instability of organic ligands under electron beam irradiation, many efforts have been made to obtain high-resolution TEM images or atomic-resolution TEM images [30,31,32]. Mayoral et al. reported that atomic observations of Zn-MOF-74 can be obtained using Cs-corrected scanning transmission electron microscopy (STEM) [31]. Zhu et al. used a direct-detection electron-counting camera to observe the local structures of an MOF (ZIF-8) with high precision, and those atomic-resolution TEM images can provide a new insight into the self-assembly mechanism of ZIF-8 [32]. In this paper, octahedral MnO/C nanocomposites were synthesized by heat treatment of Mn-MIL-100 precursors at 600 °C. MnO nanocages with a diameter of approximately 20 nm are observed in the MnO/C nanocomposites fabricated using advanced aberration-corrected electron microscopy.

## 2. Materials and Methods

### 2.1. Synthesis of MnO/C Nanocomposites

Mn-MIL-100-derived MnO/C nanocomposites were fabricated according to the following procedures: (1) adding 50 mL ethyl alcohol in a beaker; (2) dissolving 1.222 g 1,3,5-BTC (trimeric acid, 1,3,5-benzenetricarboxylic acid) in ethyl alcohol to form solution A; (3) adding 24 mL ethyl alcohol in a beaker followed by adding 2.174 g manganese nitrate (Mn(NO_3_)_2_·4H_2_O) to form solution B; (4) adding solution B to A to form a mixture; (5) heating the mixture at 120 °C for 12 h in a vacuum-drying oven; (6) centrifuging the mixture to obtain precipitation after the mixture had cooled down to room temperature; (7) washing and centrifuging the precipitation with ethyl alcohol three times; (8) drying at 60 °C for 12 h in a vacuum-drying oven to obtain Mn-MIL-100; and (9) heating the as-prepared Mn-MIL-100 precursor at 600 °C for 2 h in a tube furnace with a heating rate of 3 °C/min to obtain MOF-derived MnO/C nanocomposites with regular octahedral morphology.

### 2.2. Characterization

X-ray diffraction (XRD) patterns were collected on a Rigaku D/max 2500 PC X-ray diffractometer (Rigaku, Tokyo, Japan) with Cu K_α_ radiation source (λ = 0.15406 nm). Scanning electron microscope (SEM) images were conducted on a S-4800 field-emission scanning electron microscope (Hitachi, Tokyo, Japan). Transmission electron microscopy (TEM) images, high-resolution TEM images (HRTEM), electron diffraction pattern (EDP) and high-angle annular dark-field scanning transmission electron microscopy (HAADF-STEM) images were taken in an aberration-corrected ETEM (FEI-Titan G2, 300 kV, Thermo Fisher Scientific, San Francisco, CA, USA). Nitrogen adsorption–desorption was determined on an ASAP-2020e system analyzer (Micromeritics, Norcross, GA, USA) at 77 K to obtain the specific area and pore volume for the MOF precursor and MOF-derived MnO/C nanocomposites. Fourier-transform infrared spectroscopy (FT-IR) was conducted on a Bruker Vector 22 FT-IR spectrometer (Bruker, Berlin, Germany) to obtain the functional groups of the H_3_BTC, the as-prepared Mn-MIL-100precursor and Mn-MIL-100-derived MnO/C nanocomposites. To obtain carbon signals in MnO/C nanocomposites, a Raman spectrum was run on a Renishaw Invia Raman spectrometer (Renishaw, Gloucestershire, UK).

### 2.3. Electrochemical Characterization

In a three-electrode system with a BTS-5V 10 mA system and a 1 mol L^−1^ Na_2_SO_4_ solution, electrochemical measurements were tested. The Mn-MIL-100-derived MnO/C electrode was prepared by grinding the activated material with acetylene black and polyvinylidene fluoride (PVDF) with a mass ratio of 70:20:10. The mass loading of the activated materials on the current collector was approximately 3 mg cm^−2^. The CHI 660E electrochemical workstation (Shanghai Chenhua, Shanghai, China) was used for cyclic voltammetry (CV) and electrochemical impedance spectroscopy (EIS).

## 3. Results and Discussion

### 3.1. Structure Characterization

**Figure 1:**
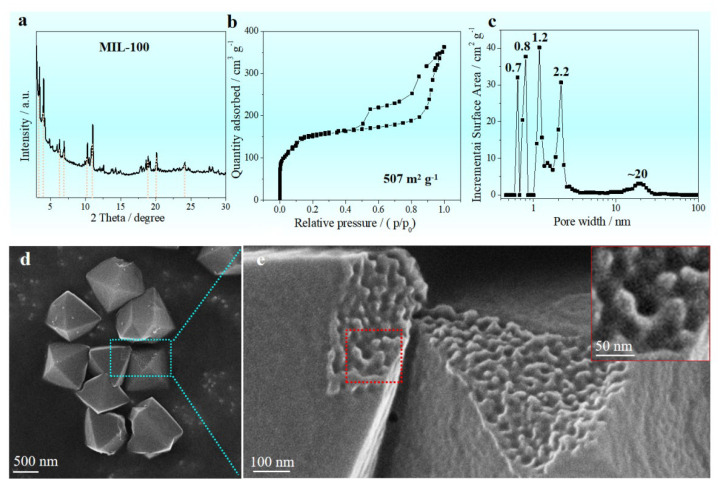
Structure characteristics of the as-prepared Mn-MIL-100 precursor. (**a**) XRD; (**b**) Nitrogen adsorption–desorption isotherms indicating a feature of microporous materials; (**c**) pore size distribution curve indicating a hierarchical pore structure; (**d**) SEM indicating octahedral morphology; (**e**) amplified SEM acquired from the blue box in (**d**) indicating the natural fracture surface of the as-prepared Mn-MIL-100 precursor with inset SEM image acquired from the red box in (**e**).

Figure 1a shows the XRD pattern of the Mn-MIL-100 precursor. Marked with vertical colored lines, the position of those Bragg peaks on the XRD pattern of the as-prepared Mn-MIL-100 precursor matches well with the XRD pattern of Mn-MIL-100 reported by Reinsch, indicating a very similar framework structure corresponding to the zeotypic MTN network, where the supertetrahedra (ST) replace the tetrahedral nodes [14]. In addition, the framework structure of the as-prepared Mn-MIL-100 precursor is also similar to the Cr-MIL-100 [29]. G Férey et al. reported a combined chemistry–simulation method to obtain MIL-100 materials, where the simulated pattern of the hybrid structure with zeotypic MTN-type matched well with the targeted experimental pattern of Cr-MIL-100, and the MOF exhibited a crystalline structure with a giant cubic cell (volume approximately 3.8 × 10^5^ Å^3^) [29].

The surface areas and porosity of the as-prepared Mn-MIL-100 precursor was examined by nitrogen adsorption–desorption measurement (Figure 1b,c). The Mn-MIL-100 precursor fabricated reveals a feature of microporous materials, as shown in Figure 1b. The Brunauer–Emmett–Teller (BET) isotherm curve gives a specific surface area of 507 m^2^ g^−1^ and total pore volume of 0.190 cm^3^ g^−1^. Micropores (0.7 nm, 0.8 nm and 1.2 nm) and mesopores (2.2 nm and 20 nm) can be detected in the as-prepared MOF precursor (Figure 1c), indicating a hierarchical pore structure. A uniform octahedral structure with a side length of roughly 1 μm is evident in the morphology of the manufactured Mn-MIL-100 precursor (Figure 1d). Figure 1e shows the amplified SEM image acquired from the blue box in d, indicating a rough and uneven surface of the natural fracture surface. The diameter of the cavity in the as-prepared Mn-MIL-100 precursor is approximately 20 nm, which matches well with the pore of 20 nm in Figure 1c.

**Figure 2:**
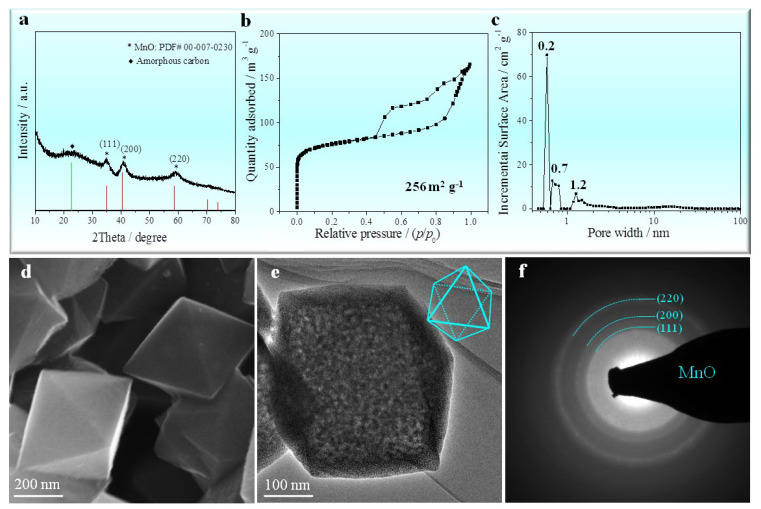
Structure characteristics of the MOF-derived octahedral MnO/C nanocomposites. (**a**) XRD; (**b**) nitrogen adsorption–desorption isotherms; (**c**) pore size distribution curve; (**d**) SEM indicating octahedral morphology; (**e**) TEM of a single octahedral MnO/C; (**f**) EDP corresponding to (**e**) showing a polycrystalline structure of the octahedral MnO/C.

Figure 2a shows the XRD pattern of the MOF-derived octahedral MnO/C nanocomposites. The XRD pattern reveals signals of a cubic structure MnO (Standard card JCPDS No. 00-007-0230) and amorphous carbon. Those peaks at 35°, 41°, and 58° correspond to the (111) (200) and (220) planes, respectively, of the cubic MnO with space group of Fm-3m (225). By measuring nitrogen adsorption–desorption at 77 K, the specific surface area and porosity of the MOF-derived octahedral MnO/C nanocomposites were also found (Figure 2b,c). The BET isotherm curve of as-prepared octahedral MnO/C gives a specific surface area of 256 m^2^ g^−1^, significantly lower than that of the Mn-MIL-100 precursor (507 m^2^ g^−1^). Although the specific surface area of as-prepared octahedral MnO/C is lower than that of Mn-MIL-100 precursor, its total pore volume increases to 0.245 cm^3^ g^−1^, approximately 29% higher than that of the precursor (0.190 cm^3^ g^−1^). When compared with the Mn-MIL-100 precursor, MOF-derived octahedral MnO/C nanocomposites have micropores around 0.2 nm, 0.7 nm and 1.2 nm (Figure 2c). Mesopores around 2.2 nm, 2.7 nm and 20 nm observed in the MOF precursor can be detected in the MnO/C nanocomposites (Figure 1c). The morphology of the MnO/C nanocomposites formed from MOFs is depicted in Figure 2d, which take a smaller regular octahedral shape with a length of about 0.5 μm. However, the MOF-derived octahedral MnO/C nanocomposites do not contain any collapsed facets. TEM (Figure 2e) and the electron diffraction pattern (EDP) (Figure 2f) of a single octahedral MnO/C indicate a polycrystalline structure, as confirmed by the electron diffraction rings corresponding to the (111), (200), and (220) planes respectively, which coincide well with those Bragg peaks on the XRD patterns (Figure 2a).

**Figure 3:**
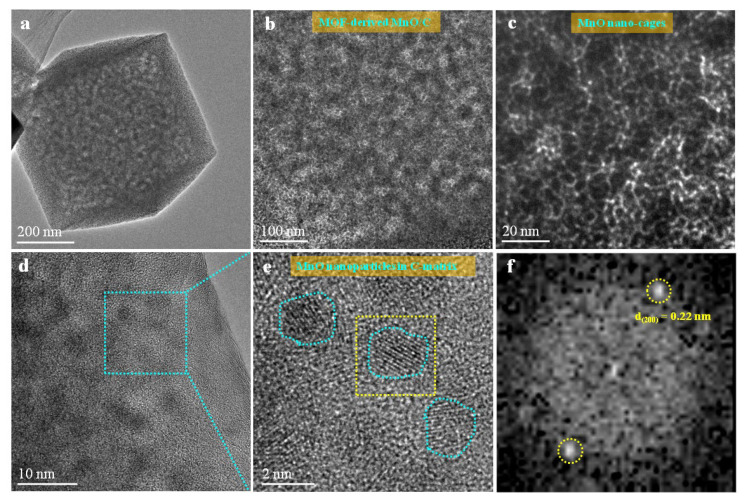
HAADF-STEM images and HRTEM images of MOF-derived MnO/C nanocomposites. (**a**) TEM images of the MnO nanocage; (**b**,**c**) HAADF-STEM; (**d**) HRTEM; (**e**) amplified image of the blue-box region indicating the MnO nanoparticles embedded in C-matrix; (**f**) FFT image shows the crystalline structure of MnO with sharp diffraction spots.

To further study the phase constituent of the MOF-derived octahedral MnO/C nanocomposites, we characterized the microstructures of the as-prepared MnO/C nanocomposites by high-angle annular dark-field scanning transmission electron microscopy (HAADF-STEM). The HAADF-ETEM image (Figure 3a) indicates the morphology of a single octahedral MnO/C. Figure 3b shows a mosaic structure with MnO nanocrystals uniformly embedded in a carbon matrix. Figure 3c shows that MnO has a three-dimensional nanocage topology structure, which was self-assembled by MnO nanocrystals of about 1–2 nm in diameter. Figure 3d is the HRTEM image of the MOF-derived octahedral MnO/C nanocomposites, demonstrating nanoscale MnO particles (~2 nm) embedded with high density in a carbon matrix homogeneously. Figure 3e is the amplified HRTEM image acquired from the blue-box region indicating that the nanoscale MnO particles embedded in C-matrix are single crystal structure, which is confirmed by the corresponding fast Fourier transform (FFT) (Figure 3f).

**Figure 4:**
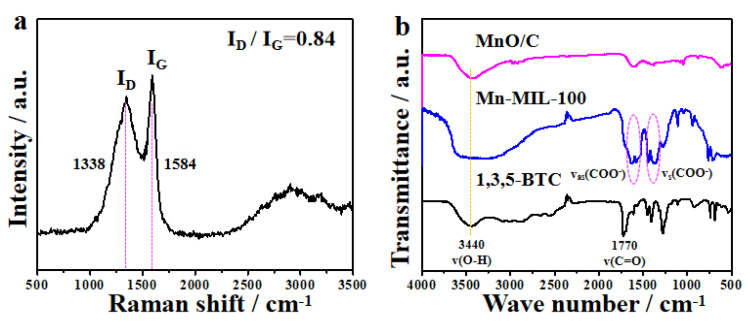
(**a**) Raman spectrum of MOF-derived MnO/C nanocomposites and (**b**) FT-IR spectra of the initial raw material 1,3,5-BTC, as-prepared Mn-MIL-100 precursor and MOF-derived octahedral MnO/C nanocomposites.

The Raman spectrum of the MOF-derived octahedral MnO/C nanocomposites (Figure 4a) exhibits two broad peaks in the range of 1200–1700 cm^−1^: D-band (1338 cm^−1^, disordered graphite) and G-band (1584 cm^−1^, crystalline graphite). The ratio of area of D-band to G-band is 0.84, indicative of a higher degree of ordered graphite. In addition, the Raman spectrum also exhibits a specific signal of D + D’ peak at 2930 cm^−1^ associated with defect density, indicating that the crystalline graphite in the MOF-derived octahedral MnO/C nanocomposites is of high defect density. Unreacted organic linkers may be connected to or encapsulated within MOFs, and in order to remove these organic linkers, thorough washing is required. To illuminate the functional groups of the 1,3,5-BTC, the as-prepared Mn-MIL-100 precursor and the Mn-MIL-100-derived MnO/C nanocomposites, FT-IR spectra were obtained on a Bruker Vector 22 FT-IR spectrometer (Figure 4b). The FT-IR spectrum of the as-prepared Mn-MIL-100 precursor reveals a totally different curve from that of the FT-IR spectrum of the initial raw material of 1,3,5-BTC, indicating that the residual acid was removed from the Mn-MIL-100 precursor thoroughly.

### 3.2. Formation Mechanism

**Figure 5:**
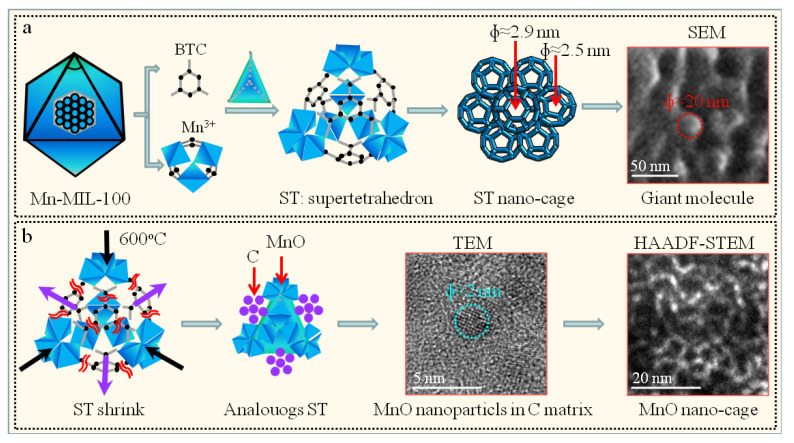
Schematic mechanism for the fabrication of as-prepared Mn-MIL-100 precursor and Mn-MIL-100-derived MnO/C nanocomposites. (**a**) Fabrication process of Mn-MIL-100 precursor, with the SEM image acquired from Figure 1e; (**b**) formation mechanisms of Mn-MIL-100-derived MnO/C nanocomposites, with the HAADF-STEM image acquired from Figure 3c.

The formation mechanism of the MOF-derived MnO/C nanocomposites with a regular octahedral morphology is schematically demonstrated in Figure 5. During the preparation process, 1,3,5-BTC acts as the trimeric building unit of the MIL-100, which is configured by the supertetrahedron (ST) with inorganic bricks as the corners and tricarboxylate anions as the faces [14,29]. As a result, Mn-MIL-100 with zeotypic MTN framework structure are obtained. In addition, two types of cavities are fabricated from the three-dimensional nanocages of the ST, the pentagonal windows and hexagonal apertures, and two types of pores are obtained with a diameter of approximately 2.5 nm and 2.9 nm, respectively. Those MIL-100 fabricated reveal similar lattice parameters for Mn-MIL-100 (7.3299 nm) and Cr-MIL-100 (7.2906 nm), respectively [14,29], and the resulting MIL-100 framework have a giant cubic cell with volume of approximately 380 nm^3^ [29]. In this paper, a very similar XRD pattern with framework structure corresponding to the zeotypic MTN network was obtained for the as-prepared Mn-MIL-100 precursor (Figure 1a), indicating similar lattice parameters and cell volume to that reported by Reinsch et al. [14]. Interestingly, spherical particles with a diameter of approximately 20 nm were observed on the natural fracture surface of as-prepared Mn-MIL-100 precursor (Figure 5a), indicating that the building units of the Mn-MIL-100 precursor are the spherical particle, a giant molecule self-assembled by the three-dimensional ST nanocages.

Note that the 1,3,5-BTC was decomposed and carbonized when the as-prepared Mn-MIL-100 precursor was heated to 600 °C, resulting in the shrink or wrinkle of the ST. It can be hypothesized that analogous ST units inheriting from the as-prepared Mn-MIL-100 precursor exist in a transitional state with manganese ion as the corners and carbon as the faces (Figure 5b). According to the phase diagram of C-Mn, Mn has no solubility in C at 600 °C, resulting in slight deviation of carbon from the faces of ST. The porous carbon-coated shell was wrapped around the MnO core, resulting in a core–shell configuration for the ST-derived MnO/C nanoparticle. The resulting framework corresponds to the three-dimensional nanocages of the ST with zeotypic MTN structure, in which the MnO nanoparticles replace the ST nodes (Figure 5b). Our work reveals that the MnO nanoparticles exhibit nanocage structure characteristic of MOF-derived MnO/C nanocomposites, which may provide guidance towards understanding other MOFs or MOF-derived materials [33,34,35,36].

### 3.3. Electrochemical Properties

**Figure 6:**
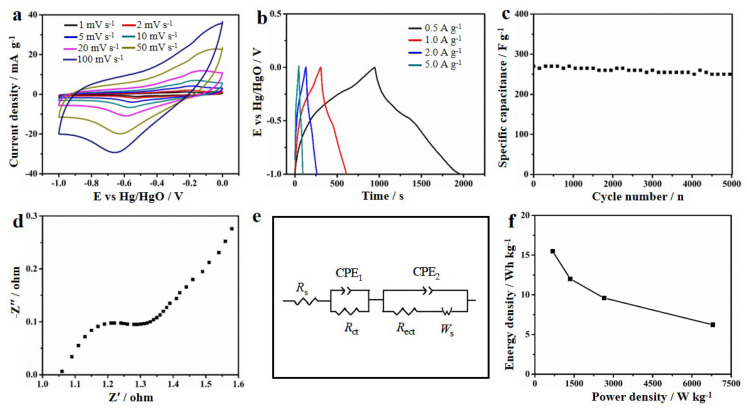
Electrochemical properties of MnO/C for supercapacitor. (**a**) CV curves; (**b**) charge–discharge curves; (**c**) cycling stability; (**d**) EIS in a high frequency region; (**e**) diagram of an equivalent circuit; and (**f**) Ragone plot of the symmetrical supercapacitor.

The electrochemical performance of MOF-derived MnO/C nanocomposites was evaluated in 1 mol L^−1^ Na_2_SO_4_ solution. The CV curves of the MnO/C electrode materials at various scan rates (1–100 mV s^−1^) with a potential range of −1.0 V to 0 V are displayed in Figure 6a. The approximate rectangular shape of the CV curves shows that the charge of the MnO/C nanocomposites varied nearly linearly with the charging potential. Instead of simple accumulating ions at the surface of the electrode, charges are stored in the MnO pseudo-capacitive electrode through surface faradic redox reactions. The electrostatic charge–discharge curves of the MnO/C electrode material at different current densities are given in Figure 6b. The MnO/C electrode exhibits an excellent high-rate discharge ability with high specific capacitance of 421, 359, 324 and 270 F g^−1^ at a current density of 0.5, 1, 2 and 5 A g^−1^, respectively. In addition, the MnO/C electrode exhibits excellent cycle stability, i.e., a high capacitance retention of 94% after 5000 cycles (Figure 6c).

Figure 6d shows the EIS of the MnO/C electrode materials. The semicircle in the high-frequency area exhibits an electrochemical reaction’s impedance. Utilizing ZView electrochemical impedance software’s least-square approach, the impedance spectra were fitted with an analogous circuit model (Figure 6e, *R*_ct_ means the electrochemical reaction’s charge transfer resistance). The fitted results show that the *R*_ct_ of MnO/C nanocomposites is about ~0.1 Ω.

To further evaluate the MnO/C electrode material, an asymmetric MnO/C//AC supercapacitor was assigned. MnO/C nanocomposites and activated carbon are used as positive and negative electrode materials (Figure 6f), respectively. The results of the symmetrical supercapacitor show that the specific energy density is 15.5 Wh kg^−1^ at a power density of 680 W kg^−1^ and the highest power density is 6800 W kg^−1^ at an energy density of 6.2 Wh kg^−1^, corresponding to Mn-based electrode materials (Figure 6f). These positive electrochemical results can be attributed to three factors: the unique nanocage structure that can restrain the volume expansion effectively, the embedded single particle structure that can suppress the agglomeration, and the 3D porous carbon network’s structure that can improve the conductivity of the MnO/C nanocomposites. Table 1 summarizes the previously investigated capacitance and cycling stability of mannite-based oxide electrode materials. [37,38,39,40] Clearly, our MnO/C electrode has both high capacitance and good cycle stability.

The as-prepared Mn-MIL-100-derived octahedral MnO/C nanocomposite electrode has high values in terms of specific capacitance, cyclic stability, as well as power density and energy density. High specific surface area (256 m^2^ g^−1^) and hierarchical pore structure with high total pore volume (0.245 cm^3^ g^−1^) affect the capacitance of the porous carbon materials in the MOF-derived octahedral MnO/C for EDLCs. In addition, the nanocage structure, the nanoscale MnO single crystal, as well as the high density of MnO nanoparticles embedded in a carbon matrix homogeneously affect the capacitance of the MnO materials for pseudo-capacitors.

## 4. Conclusions

In conclusion, utilizing Mn-MIL-100 as a precursor, high-density MnO nanoparticles with a single crystal structure were in situ synthesized. The XRD pattern of the produced Mn-MIL-100 precursor matches the framework structure of the zeolite MTN network. Spherical particles with a diameter of approximately 20 nm were observed on the natural fracture surface of the as-prepared Mn-MIL-100, indicating that the building units of the Mn-MIL-100 precursor are the spherical particle, a giant molecule self-assembled by the three-dimensional ST nanocages. MnO nanocages with a diameter of around 20 nm were identified in the Mn-MIL-100-derived MnO/C nanocomposites using HAADF-STEM images. The Mn-MIL-100-derived octahedra MnO/C nanocomposite used as a supercapacitor electrode exhibited high values.

## Figures and Tables

**Table 1:** Summary of recent progress in manganese-based oxide electrode materials.

Electrode Material	Capacity Current Density	Cycle Performance Current Density	Ref.
MnO/rGO	51.5 F g^−1^/0.5 A g^−1^	15,000 cycles/0.5 A g^−1^	[37]
MnO_x_/CCNF	271 F g^−1^/1 A g^−1^	5000 cycles/1 A g^−1^	[38]
Mn_3_O_4_/ graphene	502 F g^−1^/1 A g^−1^	1000 cycles/5 A g^−1^	[39]
MnO_x_/C	807 F g^−1^/1 A g^−1^	2500 cycles/1 A g^−1^	[40]
MnO/C	421 F g^−1^/0.5 A g^−1^	5000 cycles/5 A g^−1^	This work

## Data Availability

Data presented in this article are available on request from the corresponding author.
